# Sensitivity of Acoustic Voice Quality Measures in Simulated Reverberation Conditions

**DOI:** 10.3390/bioengineering11121253

**Published:** 2024-12-11

**Authors:** Ahmed M. Yousef, Eric J. Hunter

**Affiliations:** Department of Communication Sciences and Disorders, University of Iowa, Iowa City, IA 52242, USA; eric-hunter@uiowa.edu

**Keywords:** voice metrics, simulated room acoustics, sensitivity, speech acoustics, reverberation

## Abstract

Room reverberation can affect oral/aural communication and is especially critical in computer analysis of voice. High levels of reverberation can distort voice recordings, impacting the accuracy of quantifying voice production quality and vocal health evaluations. This study quantifies the impact of additive simulated reverberation on otherwise clean voice recordings as reflected in voice metrics commonly used for voice quality evaluation. From a larger database of voice recordings collected in a low-noise, low-reverberation environment, voice samples of a sustained [a:] vowel produced at two different speaker intents (comfortable and clear) by five healthy voice college-age female native English speakers were used. Using the reverb effect in Audacity, eight reverberation situations indicating a range of reverberation times (T20 between 0.004 and 1.82 s) were simulated and convolved with the original recordings. All voice samples, both original and reverberation-affected, were analyzed using freely available PRAAT software (version 6.0.13) to calculate five common voice parameters: jitter, shimmer, harmonic-to-noise ratio (HNR), alpha ratio, and smoothed cepstral peak prominence (CPPs). Statistical analyses assessed the sensitivity and variations in voice metrics to a range of simulated room reverberation conditions. Results showed that jitter, HNR, and alpha ratio were stable at simulated reverberation times below T20 of 1 s, with HNR and jitter more stable in the clear vocal style. Shimmer was highly sensitive even at T20 of 0.53 s, which would reflect a common room, while CPPs remained stable across all simulated reverberation conditions. Understanding the sensitivity and stability of these voice metrics to a range of room acoustics effects allows for targeted use of certain metrics even in less controlled environments, enabling selective application of stable measures like CPPs and cautious interpretation of shimmer, ensuring more reliable and accurate voice assessments.

## 1. Introduction

Previous studies demonstrated the utility of computer analysis of human voice as a convenient, accessible tool to assess vocal production quality and vocal health [1,2,3,4] compared to other assessment methods, such as laryngeal imaging and aerodynamic analysis [5,6,7,8]. When accurate acoustic voice measurements from voice samples are extracted, these can support the diagnosis and treatment of voice disorders as well as evaluate voice quality [9,10,11,12,13,14]. For example, previous studies have effectively evaluated voice with metrics such as jitter, shimmer, harmonic-to-noise ratio (HNR), and smoothed cepstral peak prominence (CPPs) [15,16,17]. The reliable and accurate extraction of voice metrics from the voice recording’s signal is crucial for the effective assessment of voice quality. But, the reliability of these acoustic voice metrics depends on many factors, such as recording equipment (e.g., microphone type, mic preamp, analog-to-digital hardware) or room conditions (e.g., reverberation, noise levels, types of noise) [18,19,20,21,22]. Not documenting or controlling for these factors reduces the confidence in calculated acoustic voice parameters. Not accounting for or controlling recording devices and room acoustics reduces the reliability of the calculated acoustic voice parameters.

Previous studies have examined how various environmental recording conditions, such as different types of recording equipment and recording quality, affect the reliability of acoustic voice parameters. For example, background noise at a signal-to-noise ratio (SNR) below 30 dB significantly impacts the reliability of acoustic voice quality measurements, such as jitter and shimmer [19,21]. Additionally, research has demonstrated that different microphone types significantly influence clinically relevant acoustic voice parameters, such as jitter, shimmer, and HNR, which, in turn, affect voice assessment outcomes [18,20,22,23,24]. While numerous papers have investigated the effects of room background noise and recording equipment on acoustic voice measurements, research on the impact of room reverberation remains relatively scarce in the literature. Studies examining voice production in physical rooms with different reverberation times found significant differences in voice quality measures [18,25]. In these cases, the room’s effect was highly influenced by the speaker’s perception of and response to the room’s reverberation, which, in turn, influenced voice production. Yet the recorded voice would also contain some degree of actual room reverberation, though the extent of this impact remains unknown. In addition to speaker adjustments to environmental conditions that typically affect the signal, previous studies often combine reverberation with background noise, making it difficult to isolate the specific effect of reverberation [18,25]. Another limitation is that research mainly focuses on the impact of reverberation only on a narrow range of acoustic voice measurements used to assess vocal health and voice quality. 

This study aims to add to our knowledge and address the previous literature gaps by using simulated room effects to investigate the impact of room reverberation times on voice metric estimation—ruling out speaker responses to the varying room conditions. Therefore, steady voice productions—recorded in a low-reverberation, low-noise environment—were convolved with simulated room impulse responses covering different reverberation times to assess their impact on common acoustic voice quality measures for steady vowel analysis. We focused on the impact of simulated reverberation on jitter, shimmer, HNR, CPPs, and alpha ratio. While CPPs is recommended as a primary acoustic voice metric in voice clinics [7], the other included measures are still frequently utilized in clinical practice by healthcare professionals, highlighting the importance of studying their reliability [26,27,28]. Understanding the sensitivity and stability of these acoustic voice metrics to a range of room reverberations, without the human voice production response, allows for targeted use of certain metrics even in less controlled environments, enabling selective application of stable acoustic voice parameters. Such a result would be translatable to a range of linguistic speech studies as well as in supporting better clinical acoustic voice assessments and reliable acoustic voice markers for vocal health across diverse environmental conditions.

## 2. Methods

### 2.1. Audio Recordings 

The audio recordings used in the current study were selected from a larger dataset [29,30] and involved five college-age female native US English speakers. These participants were recorded in a controlled laboratory environment at Michigan State University. None of the participants recorded reported any communication limitations related to hearing, speech, or voice. In clinical voice settings, it is common to obtain a steady vowel sample from clients during assessment. Therefore, the recordings selected from the larger dataset included sustained vowels [a:] held as long as comfortable. Each participant repeated the steady vowels three times at two production goals: comfortable and clear/best quality. These production goals were chosen to allow for a variety of voice production impacts from the same person as well as to assess whether production style influences the reliability of acoustic voice measures under varying room conditions. Overall, clear speech—characterized by exaggerated articulation, expanded vowel space, distinct formant frequencies, longer duration, and greater intensity—enhances intelligibility and may offer greater robustness to room acoustics than comfortable speech [31,32,33]. Furthermore, while running speech was available to be used as well, steady vowels were chosen as a first step in building our understanding since they should be more robust to reverberation effects due to the minimal dynamic changes in production (future endeavors can look at effects on running speech) [34].

The voice recordings were collected in a double-walled attenuation and isolation booth with a low background noise level of 33 dBA and a minimal reverberation time of 0.05 s. A head-mounted, omnidirectional microphone (HMM; omnidirectional; Countryman B3) was positioned approximately 5 cm from each participant’s mouth. The microphone was connected to a low-noise, high-quality pre-amplifier (Millennia HV-3D) and converted using an A/D converter (RME ADI-8 DS) through REAPER, a digital audio workstation (WAV, 16-bit, 44.1 kHz). Vowel [a:] productions were repeated three times in the two production styles and individually segmented for individual production analysis (6 for each participant). After segmentation, the middle 3 s of the steady portion was selected for analysis, ensuring it was free of abrupt terminations or initial onsets or offsets and without rapid prosodic fluctuations in pitch and amplitude; this is a common process in vocal quality assessments [35]. This was confirmed through careful listening and a perceptual check of the recordings. 

### 2.2. Simulated Room Reverberation Parameters 

The recordings described above (baseline recordings from a quiet, low-reverberation environment) were mixed with eight different levels of simulated reverberation effects, ranging from very low to high reverberation times. The reverb effect in Audacity (version 2.4.1), an audio editing software, was used to simulate various levels of reverberation. This was achieved by mixing the baseline recordings with eight preset reverberation effects in Audacity, ranging from minimal reverberation in near-anechoic conditions to highly reverberant, church-like spaces. The preset parameters for each simulated room provided by Audacity (such as room size, damping, and reverberance percentage) were used as defined to facilitate reproducibility in future studies.

Reverberation time is calculated by measuring the time it takes for the sound pressure level (SPL) of an impulse sound to decrease by a specific amount using a linear extrapolation method. This involves creating a simulated impulse response, applying reverb effects, and analyzing the decay rate of the sound to estimate the total reverberation time. In this case, the freely available AURORA signal processing plugins for Audacity (https://www.aurora-plugins.com, accessed on 15 March 2024) were used [18,36,37]. This was achieved by creating a simulated impulse response for each Audacity reverb effect using a single sample impulse, applying the reverb effects, and then using the AURORA plugin to generate a report of reverberation time at different third-octave bands (125 Hz to 16 kHz). 

In this study, T20 was used for reporting reverberation time. T20 was estimated as the time it took for the sound pressure level (SPL) to decrease by 20 dB, specifically between a 5 dB and 25 dB drop, and was then used to estimate the total reverberation time for a 60 dB decrease by extrapolation. T20 (and T30, for that matter) is particularly useful in situations where the full 60 dB decay cannot be directly measured, providing a reliable approximation of the room’s reverberation characteristics. T20’s shorter decay range (compared to T30 or T60) makes it less susceptible to modeling effects and noise, ensuring more accurate and consistent results in simulated environments. In summary, T20 was chosen for its balance of precision and practicality in estimating reverberation time in this situation [38,39,40].

**Figure 1:**
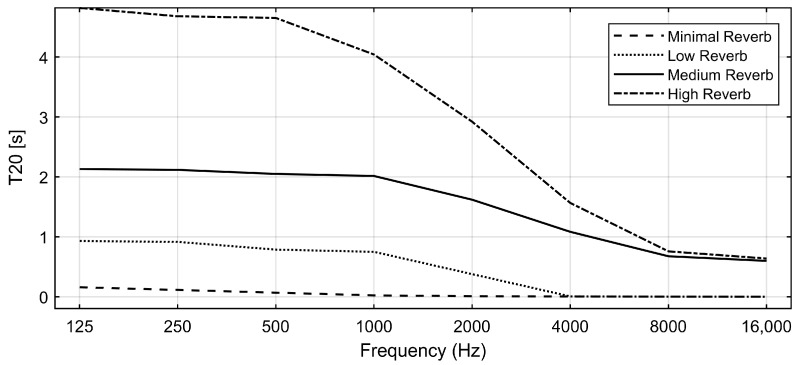
Examples of the computed reverberation time (T20) for four of the simulated room conditions across different octave band frequencies. Minimal Reverb represents a simulated anechoic chamber, while Low, Medium, and High Reverb correspond to simulated rooms with increasing levels of reverberation intensity.

Figure 1 shows an example of the calculated T20 values across different frequency bands for four levels of simulated reverberation in Audacity: Minimal (indicating a simulated anechoic chamber), Low, Medium, and High Reverb (referring to three rooms with three reverberation levels). As is common in real rooms, the simulated reverberation time decreases with higher frequencies, particularly for low, medium, and high reverberation effects. Table 1 lists the mean T20 values, averaged across all bands, for each simulated room condition and the anechoic chamber, along with the original Audacity effect names. The simulated anechoic chamber had a reverberation time (T20) of 0.004 s. While this value does not correspond to a typical room, it reflects the characteristics of treated acoustic spaces designed for minimal reverberation. This condition was included to examine the impact of the convolution/reverb process and ensure that any digital effects introduced were accounted for without adding additional reverberation.

### 2.3. Acoustic Voice Measurements

Various acoustic voice parameters, commonly used in voice evaluation, were calculated using the baseline recordings of each subject and then recalculated after applying the eight different levels of simulated reverberation. While there were many voice metrics that could have been used, we chose five commonly used to estimate voice quality in voice research and clinical voice settings and are also part of the multiparameter acoustic voice quality index (AVQI) [41,42,43]: jitter, shimmer, HNR, CPPs, and alpha ratio. These voice metrics were estimated with PRAAT software (version 6.0.13) [44]. The PRAAT software is open-source and widely used in linguistics, voice, and speech research and clinical settings, ensuring consistency and repeatability of reporting. Below is a brief description of each of these acoustic voice parameters and their relevance to voice quality assessment. 

Jitter and shimmer are time-based measures used to assess the irregularities associated with vocal fold vibration. Jitter measures cycle-to-cycle variability in the fundamental frequency, while shimmer evaluates cycle-to-cycle changes in amplitude. Higher values for both metrics suggest greater vocal fold vibration instability, indicating poorer voice quality [45]. 

HNR is another time-based key acoustic voice parameter to assess voice quality. It quantifies the ratio of harmonic (periodic) to noise (aperiodic) components in the voice signal—with higher HNR indicating a clearer, better-quality voice and lower HNR associated with increased turbulent noise, hoarseness, and vocal fold dysfunction [46,47].

The alpha ratio is a spectral-based parameter. It measures the balance between high- and low-frequency energy in the acoustic voice signal [48]. It provides insights into voice quality, with lower values indicating weaker high-frequency energy associated with hypofunctional voices and higher values reflecting stronger high-frequency energy linked to hyperfunctional voices [49,50,51,52]. 

CPPs is a spectral-based (cepstral-based) acoustic voice parameter commonly used to evaluate vocal quality and correlates with vocal health. The metric corresponds to the prominence of the harmonic structure of the voice signal, which is organized relative to the background noise caused by breathy airflow [53,54]. Higher CPPs values indicate a well-organized harmonic structure and periodic signal, reflecting better voice quality. Lower CPPs values suggest disrupted periodicity and are often associated with breathiness and roughness, making it a robust measure for assessing vocal quality [16,34].

The percent change in each voice metric due to different levels of simulated reverberation (T20 values) was calculated using the original recording measurements as the reference point. Percentage transformation was chosen to standardize measures across different scales, enabling meaningful comparisons between variables with varying units or magnitudes. By highlighting relative changes rather than absolute values, general trends and proportional relationships were highlighted in the data, which are central to the objectives of our study. Scatter plots were then created for each measure, showing the relationship between simulated reverberation time and the percent change in the measurement. A linear regression analysis was conducted to quantify this relationship, using the slope and intercept of the fitted lines to represent how each measure changes with increasing reverberation. The strength of the associations was also assessed, and the goodness-of-fit (R^2^) of each linear regression model was evaluated to ensure the reliability of the results [25]. The detailed results of this analysis are presented in the next section.

## 3. Results

Using simulated reverberation, measured in T20, the impact of reverberation levels on voice recordings was assessed in terms of five commonly used acoustic voice measures —jitter, shimmer, HNR, alpha ratio, and CPPs. The percent change in each measure was evaluated relative to its baseline (before reverberation) for two vocal styles: comfortable [a:] and clear [a:]. This section provides a detailed analysis of the results and concludes with a summary of the linear regression models (slope, intercept, and R^2^) for each measure in relation to reverberation time.

**Figure 2:**
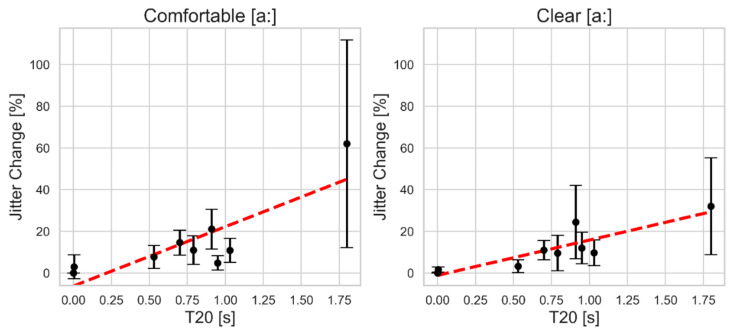
The mean and standard deviation of the absolute percent change in jitter as a function of simulated reverberation time T20 for comfortable (**left**) and clear (**right**) sustained vowel [a:] production. The red dashed line indicates the linear regression fit.

Figure 2 shows jitter as a function of T20 where the mean values for all participants are shown with error bars indicating the standard of deviation. The impact is expressed as the percent change in jitter compared to its baseline value (before adding reverberation). The *y*-axis shows the percent change, while the *x*-axis displays the reverberation time in the simulated room. The figure presents two plots, one for each vocal style: comfortable and clear. Overall, increasing reverberation time results in noticeable alterations to the original jitter values for both styles. At the lowest simulated reverberation time (0.004 s), the jitter values remain nearly identical to the original values, with only marginal changes observed. For reverberation times below 1.03 s, the difference between the two styles remains relatively small, with changes around 20%. However, at higher reverberation times, the clear style shows less jitter variability. The maximum mean changes in jitter reach about 60% in the comfortable style, compared to around 30% in the clear style at the longest reverberation time (1.80 s). Additionally, there is a noticeable increase in variability (standard deviation) in jitter change across subjects at higher reverberation levels, particularly in the comfortable style.

**Figure 3:**
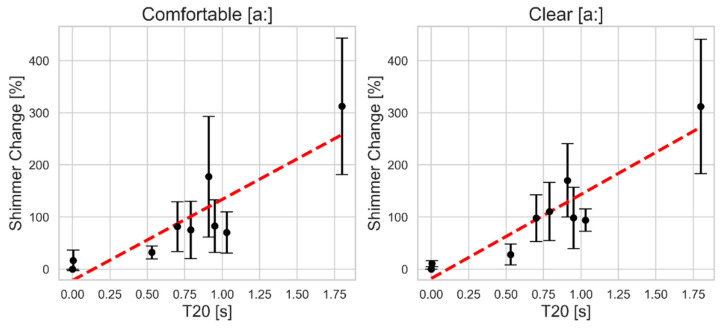
The mean and standard deviation of the absolute percent change in shimmer as a function of simulated reverberation time T20 for comfortable (**left**) and clear (**right**) sustained vowel [a:] production. The red dashed line indicates the linear regression fit.

As Figure 3 illustrates, shimmer values exhibit a much larger percent change than jitter in both vocal styles, as reflected by the steeper slope of the regression line and the expanded *y*-axis scale (0–400%). Both vocal styles display a similar upward trend with comparable mean changes and close variability across different reverberation levels. At a low reverberation time close to 0.5 s, the change in the shimmer values increases by around 40%. Between 0.7 and 1.03 s, shimmer changes nearly double from baseline, reaching close to 100%, with an anomaly at 0.91 s where the change approaches 200%. The maximum shimmer change occurs at the longest reverberation time, peaking at a 300% rise (three times the original value).

**Figure 4:**
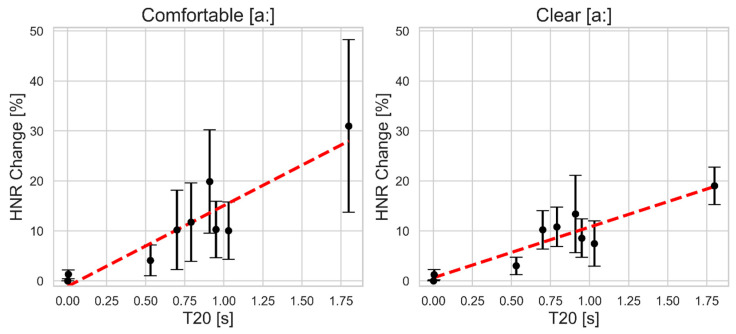
The mean and standard deviation of the absolute percent change in harmonic-to-noise ratio (HNR) as a function of simulated reverberation time T20 for comfortable (**left**) and clear (**right**) sustained vowel [a:] production. The red dashed line indicates the linear regression fit.

As shown in Figure 4, HNR is more stable than the previous two measures, with change below 20% across most common reverberation levels. Only in the comfortable style does the change reach 30% in the most reverberant case (1.80 s). Across both styles, for reverberation times between 0.7 and 1.03 s, the change fluctuates around 10%, with minimal change (approximately 3–4%) at the lowest reverberation time close to 0.5 s. Additionally, in the comfortable style, variability among subjects increases with higher reverberation levels, but in the clear style, variability remains consistent above a reverberation time of 0.6 s.

**Figure 5:**
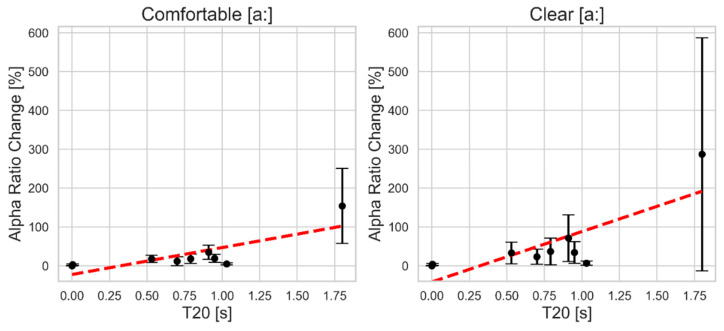
The mean and standard deviation of the absolute percent change in alpha ratio as a function of simulated reverberation time T20 for comfortable (**left**) and clear (**right**) sustained vowel [a:] production. The red dashed line indicates the linear regression fit.

As can be seen in Figure 5, alpha ratio shows similar trends for both vocal styles. At reverberation times below 1.03 s, the percent change in alpha ratio remains modest, not exceeding approximately 25% in the comfortable style. In contrast, the clear style exhibits a sharper increase, reaching around 70% at the same reverberation time. As reverberation increases further, particularly in a highly reverberant environment (T20 = 1.80 s), the percent change in alpha ratio rises significantly, approaching 150% in the comfortable style and 300% in the clear style. Excluding the maximum reverberation time, variability across subjects is relatively small in the comfortable style, while the clear style demonstrates slightly larger variability with greater standard deviation.

**Figure 6:**
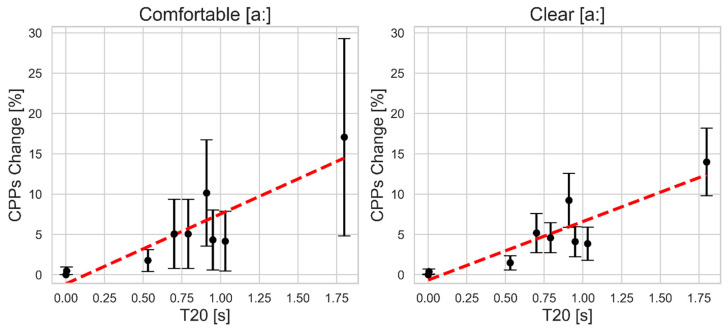
The mean and standard deviation of the absolute percent change in smoothed cepstral peak prominence (CPPs) as a function of simulated reverberation time T20 for comfortable (**left**) and clear (**right**) sustained vowel [a:] production. The red dashed line indicates the linear regression fit.

As shown in Figure 6, CPPs demonstrates a robust behavior to reverberation effects, being the least affected measure compared to the previous acoustic voice parameters. As reverberation time increases, CPPs shows a gradual upward trend, similar to the other measures. Yet, at reverberation times below 0.8 s, the percent change remains at 5% or less for both vocal styles. Even at the highest reverberation time (T20 = 1.80 s), the increase is moderate, rising to about 18% in the comfortable style and 15% in the clear style. Variability across subjects, represented by the error bars, is consistently small throughout, with the clear style showing particularly narrow margins, indicating lower variability.

Table 2 presents a summary of the linear fit equations and R^2^ values corresponding to each acoustic voice parameter included in the previous analysis, with reverberation time T20 (Rev) as the independent variable. The results give numbers to what is seen in the figures, such as shimmer change remarkably exhibiting the largest slope in both vocal styles, with a slope of 155.27 in the comfortable style and 161.28 in the clear style, indicating a strong response of shimmer to increasing reverberation. The R^2^ values for shimmer are also the highest among all measures, at 0.78 for the comfortable style and 0.86 for the clear style, suggesting a strong fit to the linear model. Alpha ratio change displays the second largest slope among the parameters. It also demonstrates the most significant difference between the two styles. The clear style has a notably steeper slope of 129.65 compared to 69.12 in the comfortable style—indicating the strong response toward reverberation in the clear style—yet both styles have the same R^2^ value of 0.63.

Jitter change follows with a moderate slope of 28.47 in the comfortable style and 16.94 in the clear style, implying greater vulnerability between the change in the jitter value and reverberation in the comfortable vocal style. The clear style shows a slightly better fit (R^2^ = 0.78) than the comfortable style (R^2^ = 0.70), and HNR and CPPs changes show smaller slopes. HNR changes exhibit a slope of 16.15 in the comfortable style and 10.14 in the clear style, with both models having high R^2^ values of 0.85 and 0.84, respectively, suggesting that the linear relationship is well-fitted in both cases with less steep slopes. CPPs changes have the lowest slopes overall (8.66 for comfortable and 7.25 for clear), with equal R^2^ values of 0.80 for both styles, indicating minimal sensitivity to reverberation impact and consistent model performance across the different vocal styles. 

## 4. Discussion

Acoustic voice analysis in isolated sound booths with high-quality recording devices ensures reliable voice evaluation. However, clinicians often collect recordings in inadequate room acoustics, either in person or remotely, impacting reliability. This study aimed to determine whether acoustic voice quality measurements are affected by changes in room conditions, specifically reverberation, and how this could potentially influence clinical assessment decisions. The present work addressed several limitations identified in previous research. Many earlier studies focused on a limited range of reverberation times or combined reverberation with other environmental factors, such as background noise [18,25]. This is because previous investigations were often limited by physical space or experimental constraints, which were addressed here by simulating a range of rooms. Furthermore, most previous studies did not control for the adjustments speakers involuntarily make in reaction to varying levels of reverberation and noise [18,23,25]. 

By isolating the effects of reverberation and simulating a broader range of conditions, this study offered a more detailed and comprehensive analysis of how reverberation alone influences five voice quality acoustic voice measures: jitter, shimmer, HNR, alpha ratio, and CPPs. These acoustic voice measurements were chosen for their reported connection to voice quality evaluation [15,16,23,55,56]. The acoustic voice parameters jitter, HNR, and alpha ratio were found to be relatively reliable measures, though only within an acceptable range of room reverberation time below T20 of around one second. Additionally, with a proper, clear vocal style, HNR and jitter became more reliable and less susceptible to adverse reverberation conditions. Shimmer, however, showed unreliability even with minimal reverberation, regardless of vocal style, suggesting caution in its interpretation. In contrast, CPPs demonstrated stability across both reverberation levels and vocal styles, making it the most reliable metric for consistent voice quality assessment. 

### 4.1. Detailed Discussion Points

The absolute percent change in the five acoustic voice parameters was examined in response to the eight simulated reverberation conditions (ranging from minimal reverb time T20 at 0.004 s to a large reverb time T20 at 1.80 s). Overall, the analysis revealed that the five acoustic voice measures demonstrated varying degrees of sensitivity to reverberation times. The most robust measure identified was CPPs, showing the least sensitivity to room reverberation among all the acoustic voice parameters. Even in a highly reverberant environment (T20 1.80 s), regardless of the vocal style, the percent change in CPPs values did not exceed 20%, indicating high stability. The linear regression model confirmed this, with CPPs showing strong R^2^ values—demonstrating a good fit—and the lowest slope reflecting its minimal vulnerability to reverberation. This high stability highlights CPPs as a reliable acoustic voice metric for assessing voice quality in varying acoustic environments and across different vocal styles in clinical settings. These findings align with a prior study, which also found that CPPs was the acoustic voice parameter least affected by room acoustics, including background noise, reverberation, and microphone type [18].

In examining the impact of reverberation on other acoustic voice measurements, jitter and HNR emerged as relatively robust parameters, demonstrating better stability than shimmer and alpha ratio. Both metrics showed behavior similar to CPPs under moderate reverberation conditions (T20 below 1.03 s), with mean changes under 20%. However, in highly reverberant conditions above 1.03 s, jitter and HNR exhibited instability, with larger percent changes. The vocal style also played a role in determining the percent change for these metrics: in the clear vocal style, both jitter and HNR did not exceed 25%, closely mirroring CPPs. In the comfortable style, however, the change climbed above 30%, indicating more susceptibility. This comparison underscores the importance of clear vocal production strategies in mitigating reverberation effects on these two measures. Most previous studies focused on the impact of background noise on the reliability of jitter and HNR [19,20,21,23,57], with findings showing jitter’s robustness compared to shimmer (aligning with our results), as well as HNR stability but within an acceptable noise level (below 43.8 dB). Few studies have examined the effects of reverberation specifically, though some reported relatively low sensitivity for jitter and higher sensitivity for HNR, often in studies that mixed reverberation with noise levels [18]. Thus, HNR and jitter can be reliable measures for evaluating voice quality in moderately reverberant clinics, especially when patients phonate in a clear vocal style, which further enhances the reliability of these measures.

Alpha ratio exhibited relatively high sensitivity to reverberation compared to HNR and jitter, as seen both visually and in the regression models, showing large slope values. However, alpha ratio displayed low percent changes at reverberation times below 1.03 s, particularly in the comfortable vocal style, indicating stability under moderate reverberation conditions. For longer reverberation times, the measure became less reliable, with more than a doubling of its original value. This finding mostly aligns with another study that showed alpha ratio’s relative robustness but under different types of environmental conditions, with moderate levels of background noise [23]. Although both studies differ in environmental conditions—background noise in that study and reverberation in the present work—the general sensitivity pattern for alpha ratio remains close. Therefore, alpha ratio can be considered a reliable measure in low-reverberation environments, but in adverse acoustic conditions, it should be interpreted carefully.

Shimmer demonstrated the highest sensitivity to increasing room reverberation compared to the other acoustic voice parameters, with this effect observed in both vocal styles. Even at relatively low reverberation (T20 around 0.5 s), shimmer values were altered by approximately 40% from their original value. Linear regression models reinforced this outcome, showing shimmer with the largest slope values among all acoustic voice metrics, suggesting its strong responsiveness to variations in room acoustics. This outcome is consistent with previous research, which indicates shimmer’s vulnerability to room acoustics, including background noise and reverberation, compared to measures like f0, alpha ratio, jitter, and CPPs [18,19,20,21,23]. This suggests that shimmer should be used with caution unless room conditions are well controlled, with minimal reverberation, to ensure reliable voice assessment. 

### 4.2. Limitations and Future Directions

As with all research, there are inherent limitations and future opportunities. In this study, room reverberation was simulated using freely available software, Audacity, and applied to clean vowel productions. While using Audacity allows for easy repeatability by others, future research could employ more advanced simulation of virtual acoustical environments (e.g., Odeon, Ramsete) and compare such simulations to actual impulse responses in real rooms (e.g., clinical rooms, classrooms, churches, or gyms). Other room acoustic factors can also be considered in future studies, along with reverberation time, such as clarity index and early decay time, which can contribute to altering the acoustic voice signal. While this study focuses on reverberation, it typically coexists with some level of room noise. Future studies should explore the controlled effects of different types of noise and eventually combine noise with reverberation. These steps are crucial for understanding the impact of room acoustics on parameters before considering the human impact of production changes within these environments. Therefore, the current study represents an essential step in this ongoing process. 

Although the acoustic voice measures studied provide insights into their reliability under varying room acoustics, their use may differ by clinical roles, such as otolaryngologists, phoneticians, and speech-language pathologists. Understanding how often healthcare professionals include acoustic voice measures in voice evaluations is crucial to contextualize their reliability. A survey could identify commonly used measures in settings like hospitals, clinics, and rehabilitation facilities and assess how these environments impact the robustness of evaluations. In addition, new measures intended for future clinical use, such as nonlinear or multiparametric approaches (e.g., dysphonia severity index, acoustic voice quality index) [53,58,59,60,61,62], should be tested for their reliability before advancing toward practical clinical applications. This would help future research expand parameters to ensure clinical relevance across diverse conditions.

Future studies could also test a range of recorded speech tasks and vocal quality. For example, recordings could include both healthy and dysphonic voices or other factors such as vocal age to support the generalizability of the findings. In this case, healthy female steady vowel recordings were used, as steady vowels are a common clinical assessment elicitation, and voice disorders are more common in females [63,64,65,66,67]. While this approach was sufficient to demonstrate the sensitivity of parameters using five samples, it limited the generalizability of findings across genders and different frequency ranges. A larger, more diverse dataset, balanced across disorders and severity and including both male and female voices, will be essential in future work to enhance robustness and applicability. Also, while the use of sustained vowels was appropriate as they are a standard task in clinical and research settings, future research should also consider the influence of room acoustics on other running speech. Regardless of these limitations and future opportunities, the present study provides practical steps necessary for understanding the effects of room acoustics on voice measures and which parameters may be more suited to the range of environmental conditions found in vocal use.

## 5. Conclusions 

In conclusion, this study provides valuable insights into the impact of simulated room reverberation on common voice quality measures, which are critical for assessing voice quality. By simulating various reverberation levels and applying them to sound-booth recordings, we eliminated extraneous environmental effects such as background noise, recording equipment, and speaker adjustments, allowing for the pure impact of reverberation to be assessed—an aspect rarely studied on its own. The findings highlight the varying reliability of acoustic voice measures under different reverberation conditions. These findings have important clinical implications for voice assessments in spaces with varying room acoustics as well as more general vocal quality assessment situations, underscoring the differential sensitivity of common acoustic voice measures to environmental conditions.

## Figures and Tables

**Table 1:** Average reverberation time (T20) values across all frequency bands for each simulated room, including the simulated anechoic chamber, generated using Audacity software (version 2.4.1).

Audacity Reverb Effect	Simulated Room	T20 (s)
Vocal I	Anechoic Chamber	0.004
Small Room Dark	Room 1	0.53
Small Room Bright	Room 2	0.70
Vocal II	Room 3	0.79
Large Room	Room 4	0.91
Medium Room	Room 5	0.95
Bathroom	Room 6	1.03
Church Hall	Room 7	1.80

**Table 2:** Linear regression models and R^2^ values for the percent change in acoustic voice parameters as a function of reverberation time T20 (Rev) for comfortable and clear style. The slope and intercept values of the fitted linear models are provided, along with the R^2^ values that indicate the goodness-of-fit for each model.

Vocal Parameter	Comfortable [a:]		Clear [a:]	
	Linear Fitted Models	R^2^	Linear Fitted Models	R^2^
Jitter change	(28.47) Rev – 6.23	0.7	(16.94) Rev – 1.12	0.78
Shimmer change	(155.27) Rev – 21.56	0.78	(161.28) Rev – 17.93	0.86
HNR change	(16.15) Rev – 1.10	0.85	(10.14) Rev – 0.63	0.84
CPPs change	(8.66) Rev – 1.12	0.8	(7.25) Rev – 0.65	0.8
Alpha ratio change	(69.12) Rev – 22.49	0.63	(129.65) Rev – 41.73	0.63

## Data Availability

The data presented in this study are available on request from the corresponding author. The data are not publicly available due to ethical restrictions.
